# Antibiotic perturbation of the murine gut microbiome introduces inter-individual susceptibility to arsenic

**DOI:** 10.1016/j.tox.2021.152798

**Published:** 2021-04-24

**Authors:** Barbara A. Roggenbeck, Lila K. Bull Chief, Seth T. Walk

**Affiliations:** Department of Microbiology and Immunology, Montana State University, Bozeman, MT, 59717, United States

**Keywords:** Arsenic, Arsenite, Arsenate, Antibiotic, Gut microbiome

## Abstract

Arsenic is a Group 1 human carcinogen and at least 200 million people around the world are exposed to unsafe levels of arsenic, predominantly through contaminated drinking water. Arsenic has also been used for hundreds, if not thousands, of years as an intentional poison due to its odorless/tasteless properties and the general lack of technology required to identify it. Both acute and chronic arsenic-related health outcomes are highly variable among similarly exposed individuals even after controlling for important factors, like host genetics, making the mechanisms underlying this often-made epidemiologic observation difficult to experimentally address and not fully understood.

Here, we describe an experimental model of arsenic exposure in C57BL/6 mice that recapitulates key aspects of inter-individuality in disease observed in humans. We show that co-administration of the antibiotic, cefoperazone, and high-level arsenic (100 ppm, inorganic sodium arsenate) results in incomplete mortality with a ratio of 60 % lethality to 40 % survival, and that survival, at least in part, depends not only on an intact microbiome but also a regulated response involved with water transport.

This work provides an experimental framework for identifying critical pathways involved in generating inter-individual variability in disease outcome following arsenic exposure.

## 1. Introduction

Exposure to arsenic promotes the onset and progression of a variety of diseases, including cancer, but well studied host factors (e.g. genetics and diet) do not fully explain disease (Antonelli et al., 2014; International Agency for Research on Cancer, 2012; Naujokas et al., 2013; Tellez-Plaza et al., 2013). As such, environmental factors are likely to contribute significantly to disease presentation. We recently showed that gut microbiome diversity, a factor already known to differ markedly between individuals, has a significant influence on arsenic toxicity (Coryell et al., 2018). We also showed that *in situ* expression of a single gene by a gut microbiome member provides significant protection to high-level (20 ppm, inorganic sodium arsenite) exposure (Wang et al., 2021). These efforts provide evidence that gut microbes and their arsenic-active metabolism(s) influence arsenic toxicity.

Rodent models are generally resistant to arsenic induced diseases, including cancer, making it difficult to study the underlying mechanisms of toxicity and carcinogenesis (Cohen et al., 2007). Reliance on a limited number of animal models and epidemiological studies has not been sufficient for identifying key aspects of arsenic toxicity, which is critical for developing prevention and treatment strategies. Furthermore, beyond using gnotobiotic or humanized mouse models, where the gut microbiome has been prospectively controlled and/or manipulated, there are few, if any, arsenic exposure models that adequately recapitulate inter-individual differences in toxicity observed in human populations (Coryell et al., 2019). The overall objective of this study was to investigate an observation made in our laboratory, where conventional C57BL/6 mice showed profound inter-individual variability in disease course and survival during acute arsenic exposure.

## 2. Methods

### 2.1. Chemicals

Inorganic arsenate (iAs^V^; sodium arsenate dibasic heptahydrate; ACS grade; ≥ 98 % purity; CAS 10048-95-0) was purchased from Sigma-Aldrich (St. Louis, MO). Cefoperazone (cefoperazone sodium salt; purity limit: 870-1015 μg/mg; CAS 62893-20-3) was purchased from Chem-Impex International Inc. (Wood Dale, IL).

### 2.2. Experimental animals

Animal experiments were approved by the Montana State University Institutional Animal Care and Use Committee. Mice were originally obtained from Jackson Laboratories (C57B/6 J) but they were bred and maintained at an American Association for the Accreditation of Laboratory Animal Care (AALAC)-accredited facility at Montana State University. Mice were housed under specific pathogen free conditions (including norovirus) in individually ventilated cages with sterilized bedding. Treatments were made fresh during all experiments and replaced 2–3 times per week. Mice were exposed to the above treatments for a pre-determined time (up to 28 days) or until pre-determined experimental or humane endpoints were met (See Supplementary Information). All experiments were conducted with veterinarian oversight and health assessment, as needed. Water consumption and weights were monitored throughout each experiment. Terminal bleeds were conducted under deep isoflurane anesthesia and ceca and spleens were collected and weighed.

For hydration experiments, WT mice were treated with cefoperazone and arsenic as described above (treatment #3). Half of the mice received daily 1 mL subcutaneous injections of 0.9 % sterile saline daily from days 4–14 of co-exposure after which no more injections were given. Water consumption and weights were monitored for the duration of the experiment. Mice were monitored for an additional 14 days or until humane endpoints were reached.

### 2.3. Blood chemistry

Blood was collected from the vena cava, and serum was isolated using serum separator tubes (BD microtainer ® blood collection tubes; Franklin Lakes, NJ). Blood and serum were pooled from 2 mice of the same sex for each sample due to low blood volume in sick mice. Serum samples were sent to IDEXX BioAnalytics (Sacramento, CA) for clinical chemistry analysis (Chem 6006 panel).

### 2.4. Statistical comparisons

All statistics were performed in GraphPad Prism (version 9.0.1, GraphPad Software, LLC). The Log-rank (Mantel-Cox) test was performed on survival data between experimental groups and pairwise hazard ratios were generated using the Mantel-Haenszel method.

## 3. Results

### 3.1. Inter-individual lethal effects due to combined antibiotic-arsenic exposure

**Fig. 1.**
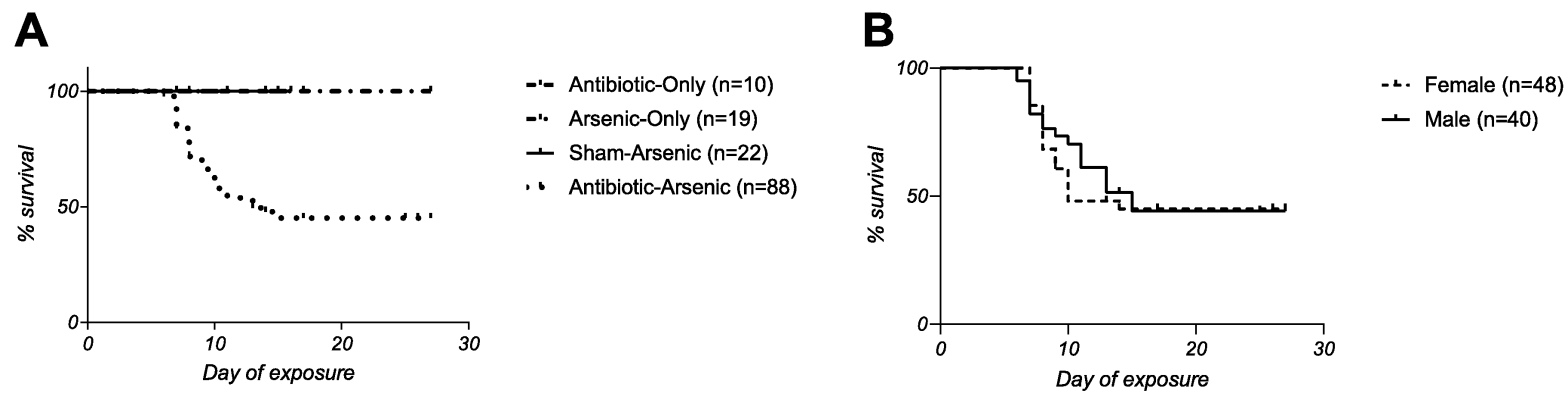
Antibiotic-associated, inter-individual susceptibility to arsenic. (A) Survival of WT C57B6/J mice exposed to the following treatments in drinking water: 1) 100 ppm iAs^V^ (arsenic-only); 2) 0.5 mg/mL cefoperazone (antibiotic-only); 3) heat-denatured cefoperazone in drinking water for 48 h prior to the addition of 100 ppm iAsV (sham-arsenic); and 4) 0.5 mg/mL cefoperazone for 48 h prior to the addition of 100 ppm iAsV (antibiotic-arsenic). Significant mortality was only observed in the antibiotic-arsenic group (p < 0.0001, Mantel-Cox test) (B) No sex differences were observed in antibiotic-arsenic group (p = 0.2400, Mantel-Cox test).

The following experimental treatment groups were considered: 1) 100 ppm iAs^V^ in drinking water (arsenic-only); 2) 0.5 mg/mL cefoperazone in drinking water (antibiotic-only); 3) heat-denatured cefoperazone in drinking water for 48 h prior to the addition of 100 ppm iAs^V^ (sham-arsenic); and 4) 0.5 mg/mL cefoperazone for 48 h prior to the addition of 100 ppm iAs^V^ in drinking water (antibiotic-arsenic). A total of 10 experiments were conducted that typically included both sexes, five mice per cage, and at least two treatment groups per experiment (Supplementary Table 1). Significant mortality, defined as reaching a predetermined humane endpoint (see Supplementary information), was only observed in the antibiotic-arsenic treatment group (Fig. 1A; p < 0.0001, Mantel-Cox Test). Mice in the antibiotic-only and sham-arsenic treatment groups showed no obvious signs of disease during exposures that lasted up to two weeks and the arsenic-only group showed no obvious signs of disease during exposures that lasted up to four weeks. In contrast, some mice in the antibiotic-arsenic group showed significant morbidity beginning as early as 4 days post-arsenic exposure and survival decreased to 42 % by 15 days post-arsenic exposure. Remarkably, mice that survived past 15 days of the exposure showed few, if any, obvious signs of disease at any point in the experimental timeline, suggesting they had somehow regulated their response to the combined effects of the antibiotic and arsenic.

Antibiotic-arsenic co-exposure was associated with an average hazard ratio of 4.2 when compared to the other groups, meaning that mice in this treatment group were at least four times more likely to reach a humane endpoint compared to mice in other groups. More mice were considered in this group (n = 88) because mortality between cages was variable. For example, at least one but not all mice required euthanasia in 79 % (11 of 14) of cages, and in only 3 instances did we observe either complete survival (n = 1 cage) or complete mortality (n = 2 cages). Such results highlight that the effect of antibiotic-arsenic treatment was highly dependent on individual factors as opposed to the C57BL/6 J host as a whole.

### 3.2. Weight loss and gross pathology associated with lethal effects

**Fig. 2.**
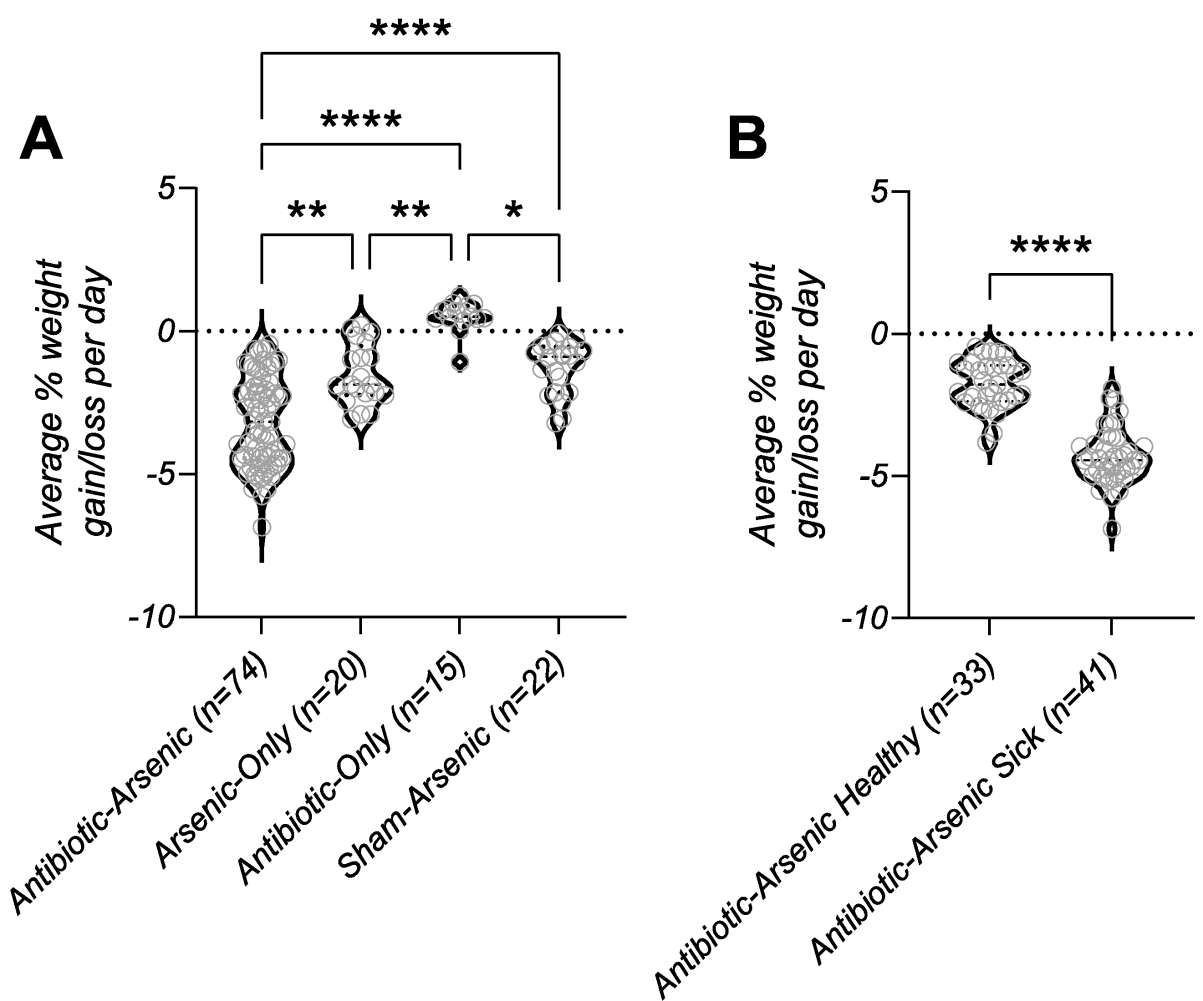
Weight loss during exposure. (A) Weight loss was summarized as the average percent (%) change per mouse per day for treatment groups described in Fig. 1. Mice in the antibiotic-arsenic treatment group lost significantly more weight than each of the other groups. (B) Healthy mice in the antibiotic-arsenic lost significantly less weight than mice in the same treatment groups that required euthanasia. *p-value < 0.05, **p-value *<* 0.01, ****p-value < 0.0001; p-values in panel A represent results of Kruskal-Wallis test with Dunn’s multiple comparisons test; p-values in panel B represent results of Mann-Whitney test.

**Fig. 3.**
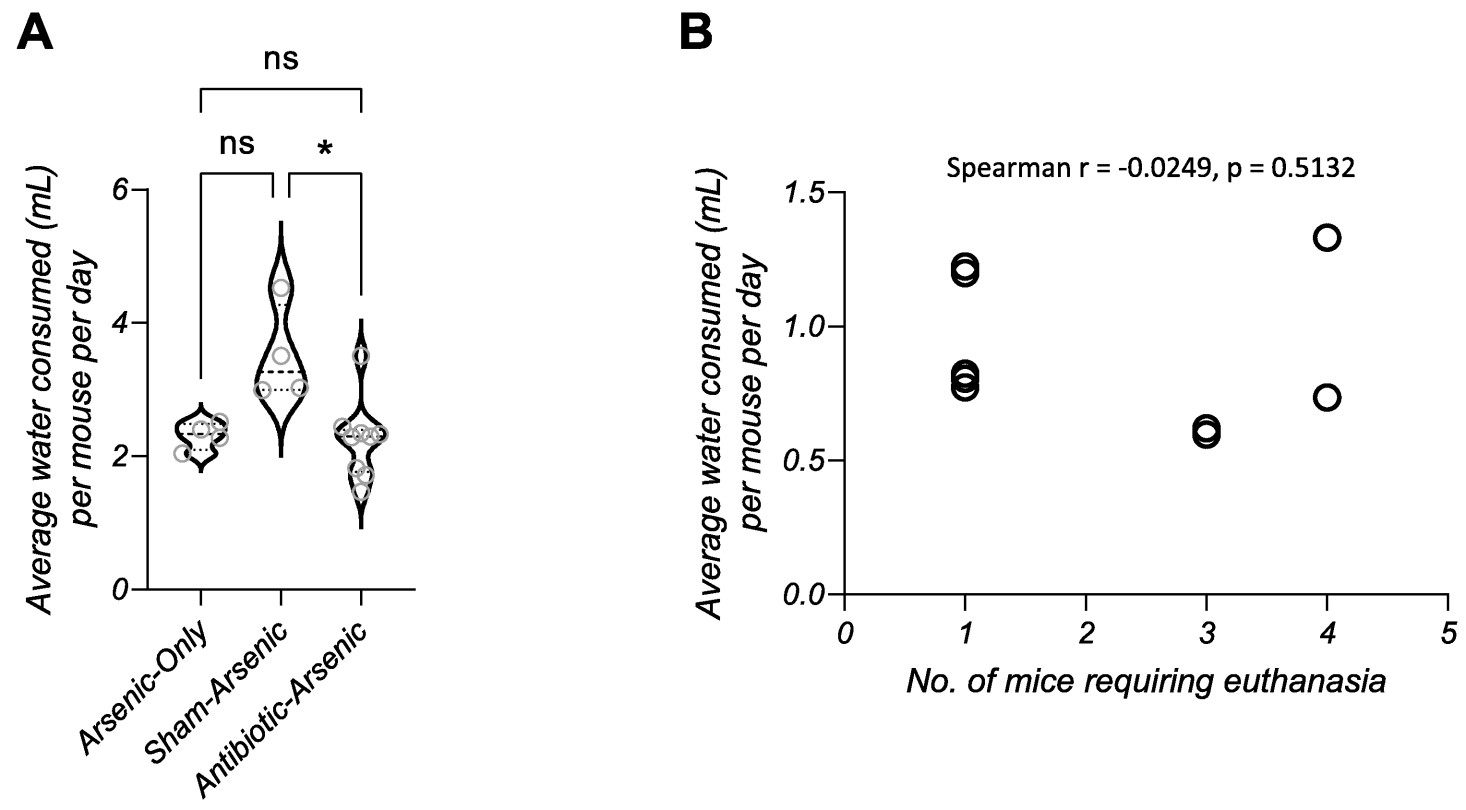
Water consumption between arsenic-exposed groups. (A) Water consumption was quantified at the cage level and summarized as the average amount of water consumed (mL) per mouse per day of exposure. Mice in the sham-arsenic group drank more water than mice in the antibiotic-arsenic group (p = 0.0226, Dunn’s multiple comparisons test), but no difference was observed in consumption between mice in the arsenic-only and antibiotic-arsenic groups. (B) The average water consumed in cages of antibiotic-arsenic mice during exposure was not correlated with the number of mice requiring euthanasia (Spearman r = −0.0249, p = 0.5132).

Females in the antibiotic-arsenic treatment group had statistically indistinguishable survival compared to males (p = 0.6756, Fig. 1B), suggesting sex was not a strong determinant of disease. Mice in the antibiotic-arsenic group did lose significantly more weight during the exposure compared to the other groups (Fig. 2A). However, mice in this group that appeared healthy lost significantly less weight compared to those requiring euthanasia (Fig. 2B). Because of the difference in weight loss, we next evaluated the hypothesis that mice requiring euthanasia drank less water during exposure, which led to severe morbidity-associated dehydration. First, we measured drinking water intake on the cage level (based on the weight change of water bottles) and calculated the average water consumption per mouse per day between the three arsenic-exposed groups (Fig. 3A). Mice in the sham-arsenic group appeared to drink the most water (median = 3.268 mL per mouse per day), which was significantly more than mice in the arsenic antibiotic group (median = 2.247 mL per mouse per day; p = 0.0226) but not mice is the arsenic-only group (2.309 mL per mouse per day; p = 0.1269). Mice in the antibiotic-arsenic and arsenic-only groups consumed almost identical amounts of water (median = 2.247 and 2.309 mL per mouse per day, respectively), suggesting that median water consumption alone did not drive the difference in disease susceptibility between these groups (antibiotic-arsenic and arsenic-only). Next, we evaluated whether drinking water intake among cages in the antibiotic-arsenic treatment was correlated with the number of mice requiring euthanasia (Fig. 3B). No correlation was found (p = 0.5132, Spearman’s r), again suggesting that water intake alone did not drive observed disease.

**Fig. 4.**
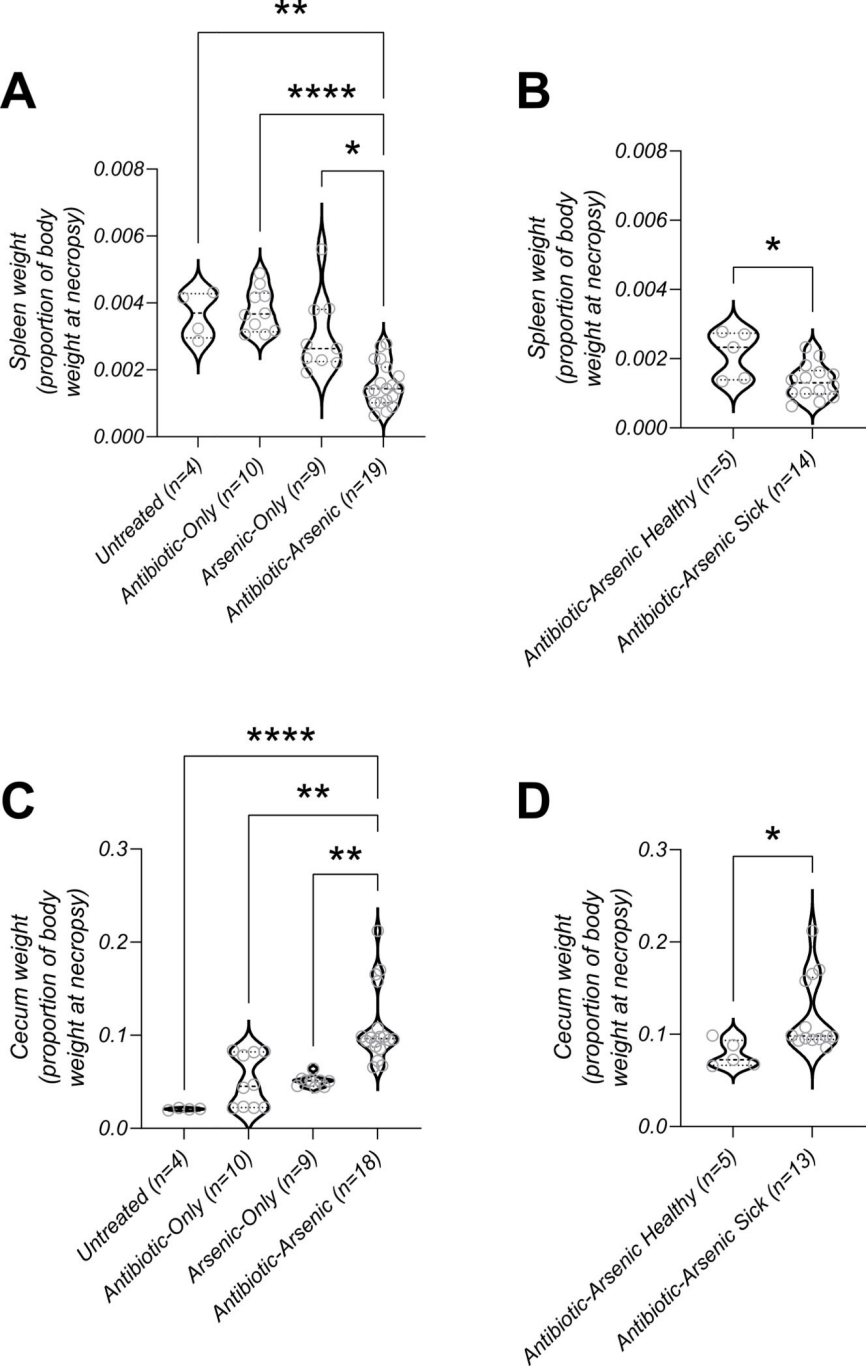
Spleen and cecum weights. (A) Spleen weights (as a proportion of body weight) were lower in mice in the antibiotic-arsenic group compared to mice in other groups. (B) Within the antibiotic-arsenic group, spleen weights in mice that required euthanasia (sick) were lower than mice that appeared healthy. (C) Cecum weights (as a proportion of body weight) were higher in mice in the antibiotic-arsenic group compared to mice in other groups. (D) Within the antibiotic-arsenic group, cecum weights in mice that required euthanasia (sick) were higher than mice that appeared healthy. *p-value < 0.05, **p-value < 0.01, ****p-value < 0.0001; p-values in panels A and C represent results of Kruskal-Wallis test with Dunn’s multiple comparisons test; p-values in panel B and D represent results of Mann-Whitney test.

**Fig. 5.**
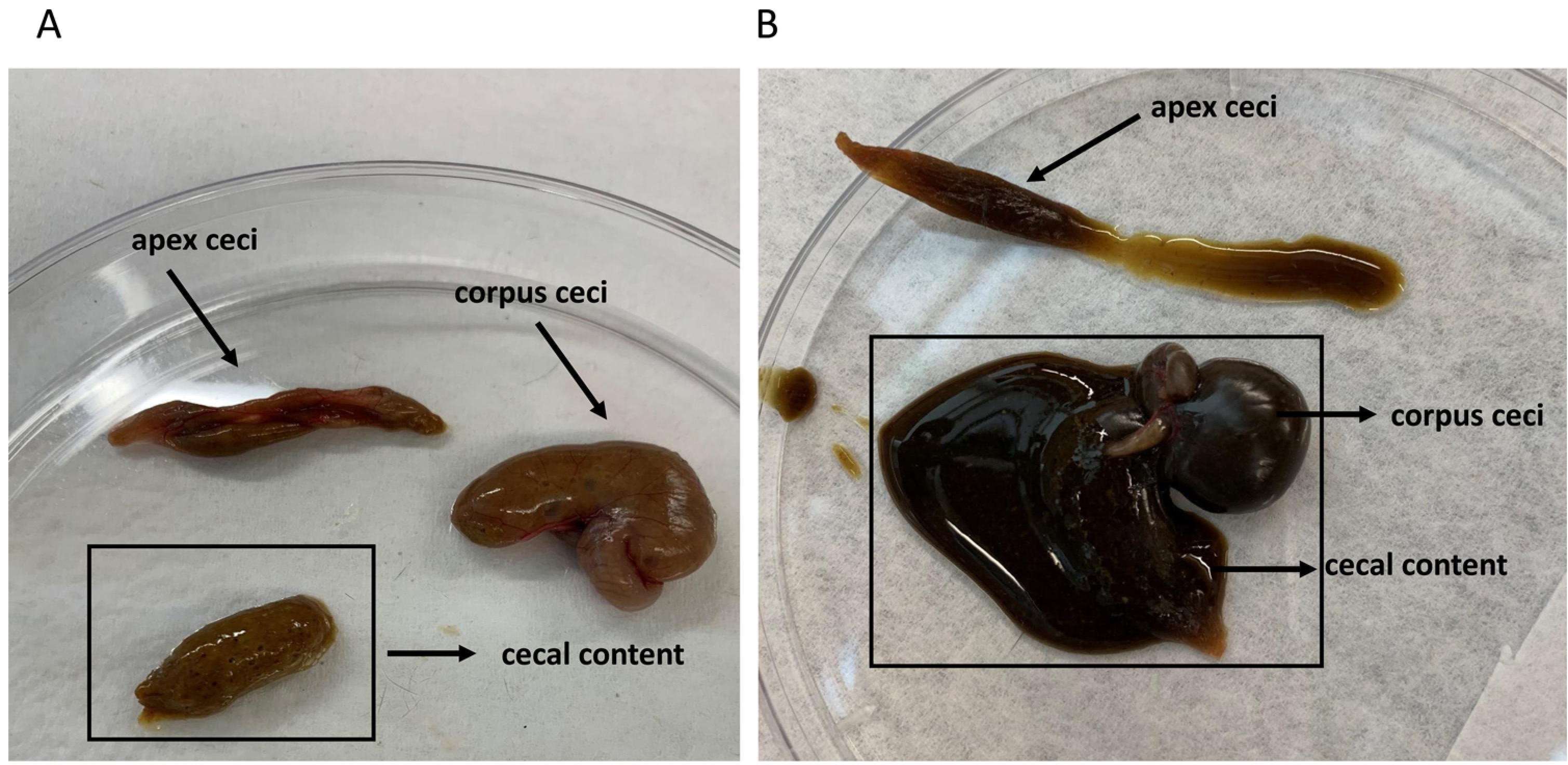
Cecal contents of antibiotic-arsenic co-exposed mice. Anatomy of cecum and contents are identified with black arrows. (A) Healthy antibiotic-arsenic mice have lighter and more viscous content compared to the (B) sick antibiotic-arsenic mice.

Inspection of internal organs at necropsy revealed that mice in the antibiotic-arsenic group had decreased spleen and increased cecum weights (Fig. 4A and C, respectively). Furthermore, mice requiring euthanasia in the antibiotic-arsenic group had smaller spleens (Fig. 4B; p = 0.0437) and larger ceca (Fig. 4D; p = 0.0194) compared to healthy mice in the same group. Cecal contents of moribund and healthy antibiotic-arsenic mice were also visibly different with healthy mice having normal looking ceca (lighter in color and more viscous) compared to the darker, more fluid-containing contents of their sick counterparts (Fig. 5). Collectively, comparisons of weight loss, water intake, and gross pathology (spleen/cecum) between the different treatment groups supported the hypothesis that some mice in the antibiotic-arsenic co-exposure group had a protective, regulated response.

### 3.3. Serum analytes associated with mortality

**Fig. 6.**
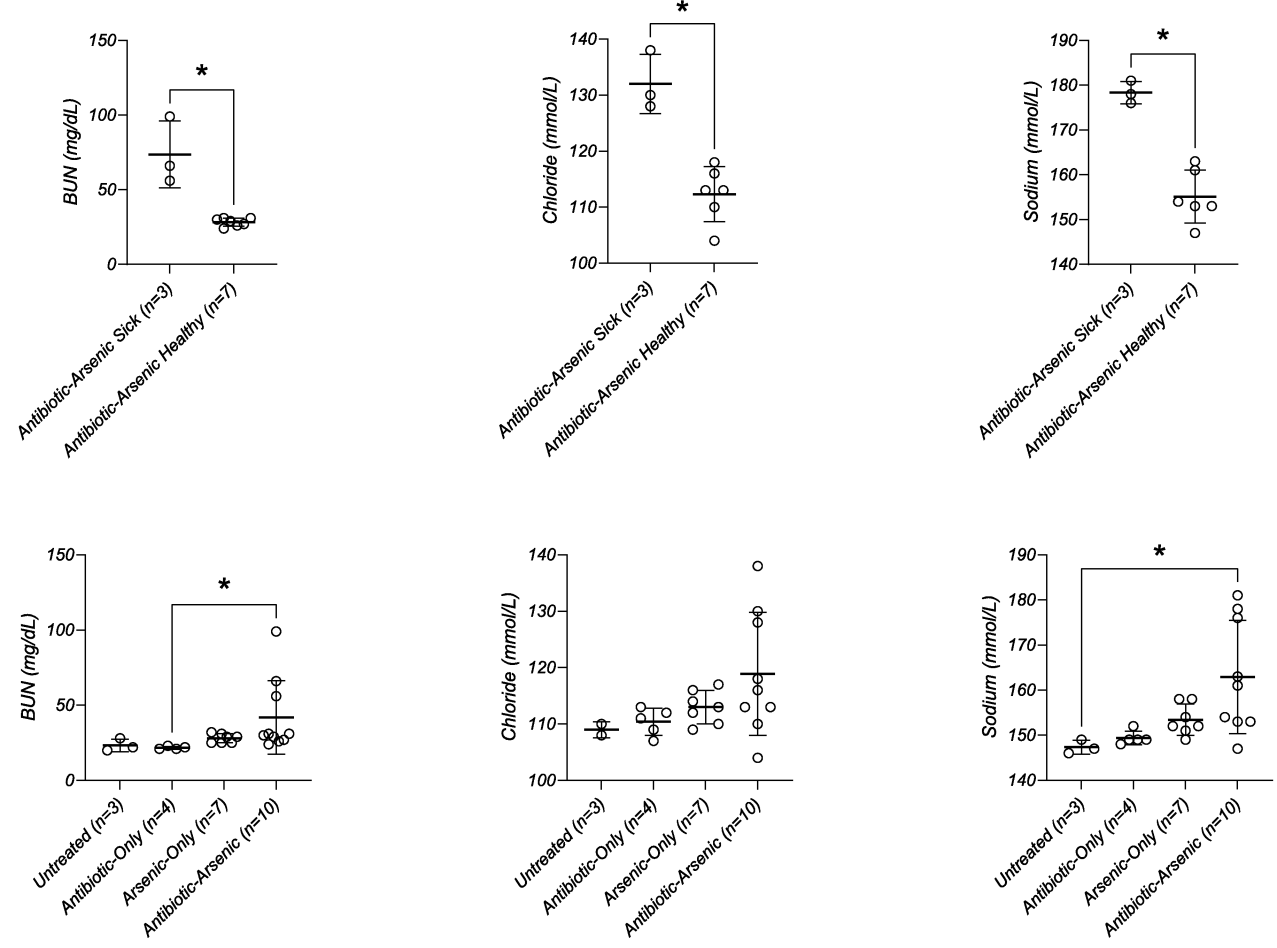
Selected serum analytes from clinical chemistry panel. (Top row) BUN, chloride, and sodium levels were elevated in antibiotic-arsenic treated mice requiring euthanasia compared to mice in the same group that showed no signs of disease. (Bottom row) Differences between all antibiotic-arsenic treated mice as other groups shown for comparison. *p-value < 0.05; p-values in top row represent results of Mann Whitney test; p-values in bottom row represent results from Kruskal-Wallis test with Dunn’s multiple comparisons test.

Clinical chemistry panels were performed to better understand and compare the overall physiologic condition of mice in different treatment groups. Blood volume was low in moribund mice of the antibiotic-arsenic group, making it necessary to pool blood from 2 mice to obtain the necessary volume for analysis. Decreased blood volume indicated that these animals were severely dehydrated. Consistent with this hypothesis, blood urea nitrogen (BUN), chloride, and sodium were significantly elevated in antibiotic-arsenic treated mice requiring euthanasia compared to healthy mice in the same treatment group (Fig. 6 top). Such differences were not always apparent in group-wise comparisons (Fig. 6 bottom), likely because unequal numbers of sick (n = 3) and healthy (n = 7) mice were compared. Regardless, these results provided strong evidence that antibiotic-arsenic treated mice requiring euthanasia were dehydrated.

### 3.4. Hydration prevents disease progression but does not reverse it

**Fig. 7.**
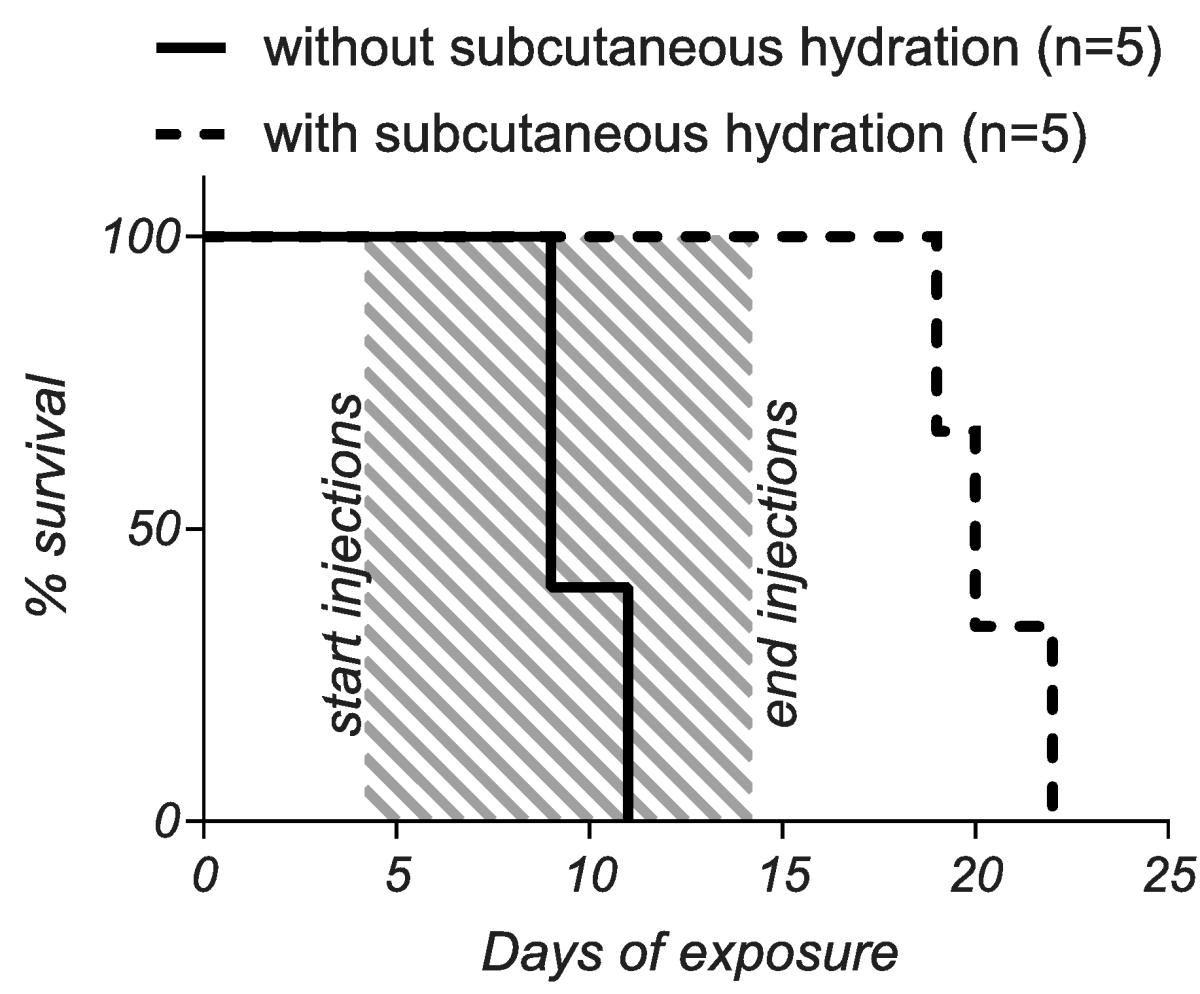
Subcutaneous hydration protects but does not cure mice. All mice were treated with antibiotic-arsenic combination and injections of saline (1 mL of 0.9 % sterile saline) were administered to only one of the groups beginning on day 4 (when the first signs of disease were present) and ending on day 14 of exposure (shaded area). Mice that received subcutaneous hydration survived significantly longer than mice that did not receive subcutaneous hydration (p < 0.0001, Mantel-Cox test), but these mice eventually succumbed to disease approximately one week after injections were stopped.

Given the above results implicating dehydration in the disease process, an experiment was performed to understand the impact of subcutaneous hydration. Two separate cages of mice began antibiotic-arsenic exposure at the same time. Disease in our model was scored visually using set criteria and ranked into three progressive phases (Supplementary Information). Fortuitously, all mice (i.e. mice in both cages) began to show signs of disease, reaching Phase 1 around day 4 of exposure. At this point, hydration was administered subcutaneously (1 mL of 0.9 % sterile saline) to mice in one cage but not the other for 10 days (i.e. days 4 through 14). As expected, mice that did not receive hydration progressed rapidly to Phases 2 and 3, requiring euthanasia between days 9 and 11 (Fig. 7). In contrast, subcutaneous hydration prevented mice from progressing past Phase 1 and once hydration was ended (day 14), mice progressed through Phases 2 and 3, requiring euthanasia approximately one week later. These results confirmed that progression of disease to the point of lethality was at least partially due to dehydration. However, hydration alone did not reverse disease, suggesting that regulation or lack thereof takes place early during exposure.

## 4. Discussion

### 4.1. Relevance to human toxicology

Identifying factors that influence the onset and progression of complex diseases is a prerequisite for the development of effective prevention and treatment strategies. Arsenicosis (arsenic-related disease) is undoubtedly complex and while important factors underlying the presentation of symptoms include the level and duration of exposure, arsenical species, human genetics, and diet, the combined influence of these factors does not fully explain disease (Cohen et al., 2013). We recently showed that the gut microbiome is likely a key determinant of arsenicosis, influencing toxicity among groups of antibiotic-treated, germ free, and gnotobiotic mice (e.g. mice hosting microbiomes from different individuals) (Coryell et al., 2018). The research presented here builds significantly on this work by describing another important influence of the gut microbiome – the introduction of inter-individual susceptibility.

Unlike our previous work that focused primarily on arsenic toxicity in the arsenic +3 methyltransferase (As3mt) deficient mouse (Drobna et al., 2009), research described here evaluated arsenicosis in conventional, C57BL/6 J mice. This is likely the most popular mouse strain used for human disease modeling and it is an inbred mouse line such that individuals should be effectively identical with respect to genetics. Mice in our experiments also had access to the same diet and built environment (cages, light cycle, etc.). Under these highly controlled conditions, the only requirement for toxicity in our experiments was the combination of antibiotic perturbation of the microbiome prior to high-level inorganic arsenic exposure. Antibiotics in general can indirectly impact host metabolism, immunity, the nervous system, and other key aspects of normal physiology through their influence on the diversity and function of microbial communities (Clemente et al., 2012; Cryan et al., 2020; Rooks and Garrett, 2016; Tang et al., 2019). Cefoperazone has been well-studied in C57BL/6 J mice, where it decreases the abundance of bacterial taxa that numerically dominate the gut of untreated animals (*Firmicutes* and *Bacteroides*) (Reeves et al., 2011); decreases the total bacterial load in the gut by ∼3 orders of magnitude (Antonopoulos et al., 2009); significantly alters bile acid levels and composition (Sun et al., 2020; Theriot et al., 2016); and opens niches for opportunistic pathogens like *Clostridioides difficile* (Jenior et al., 2018) and *Candida albicans* (Antonopoulos et al., 2009; Gutierrez et al., 2020). We hypothesized that the combination of gut microbiome perturbation and arsenic would be detrimental compared to mice treated with either antibiotic or arsenic alone, and while this was certainly the case, we did not necessarily expect to observe such interindividual variability in host response and outcome. It is worth noting again that the disease course was remarkably different between individuals in the antibiotic-arsenic treatment and bifurcated individuals into groups that showed few, if any, signs of disease and those experiencing severe morbidity to the point of humane euthanasia.

Such diversity in disease course recapitulates observations reported among victims of a very unfortunate episode of intentional arsenic poisoning in Wakayama, Japan. Of the 67 individuals that developed symptoms after ingesting a curry soup containing ≥6000 μg/g of inorganic arsenic as arsenic trioxide, four individuals aged 10–64 years died within 12 h (1 female, 3 male). According to a recent follow-up study of the event (Yamauchi and Takata, 2021), all 67 individuals experienced some sort of gastrointestinal symptom with vomiting and nausea being the most common, followed by abdominal pain and diarrhea. Importantly, some but not all poisoned individuals experienced severe, acute symptoms (skin lesions and peripheral neuropathy) and of those that did, the onset and progression of symptoms were highly variable (e.g. peripheral neuropathy in some adults had not resolved even after four years of follow-up) (Yamauchi and Takata, 2021). Another important finding of the Wakayama study was that poisoned children not only recovered more rapidly compared to adults, but also had significantly higher levels of methylated arsenic in their urine. This suggests that children were protected from high levels of arsenic exposure and it is tempting to hypothesize that at least some of this protection was due to well-known differences between the gut microbiome of children and adults (Ruan et al., 2020).

### 4.2. Why did disease present in some but not all antibiotic-arsenic treated mice?

Multiple lines of evidence support the hypothesis that some but not all antibiotic-arsenic treated mice were able to maintain normal levels of water uptake and that this was at least partially responsible for lethality. For example, we did not find evidence that antibiotic-arsenic mice drank less water compared to mice treated with arsenic alone and yet, antibiotic-arsenic mice became moribund and were severely dehydrated. Further, subcutaneous hydration halted but did not reverse disease progression in these animals, suggesting that the ability to take up water normally had already been lost. Finally, differences observed in cecal weights and visual comparison of cecal contents suggest that water uptake deficits arose in the intestine where this important process normally takes place. It is reasonable that other observed signs of disease (weight loss, anorexia, spleen weight) were inevitable downstream consequences of dysregulated water uptake but additional experiments will be required to support this hypothesis.

### 4.3. Microbiome effects on water homeostasis and future directions

Short chain fatty acid (SCFA) production by the gut microbiome is critical for water and electrolyte homeostasis in the gastrointestinal tract. Studies in rats characterizing SCFA stimulated electroneutral NaCl exchange across the apical membrane of colonocytes (Binder, 2010) suggest that this exchange mechanism results in increased fluid and electrolyte absorption in the colon (Binder, 2010; Binder and Mehta, 1989). Since cefoperazone decreases the levels of SCFAs, acetate, butyrate, and propionate, between 2- to 4-fold in the murine cecum (Guinan et al., 2019), it seems reasonable that observed differences in hydration status, weight loss, and cecal content between healthy and moribund mice in the antibiotic-arsenic group were due to dysregulation of the SCFA-driven water homeostatic mechanism. It is possible that healthy mice maintained SCFA production because the effects of cefoperazone were somehow incomplete compared to mice requiring euthanasia. Future studies are needed to quantify SCFA levels during the 48-h antibiotic pretreatment as well as the relative abundance of known SCFA-producing microbes. Follow-on experiments using gnotobiotic mice with and without SCFA production in the gut can add further evidence of causation. Ultimately, epidemiologic evidence from arsenic-exposed populations will be needed to determine whether and to what extent SCFA levels add significantly to the list of known factors underlying arsenicosis. Regardless, the research presented here represents a valuable first step in evaluating inter-individuality of arsenic toxicity and disease outcomes and provides the field with a new model to investigate this important aspect of disease.

## Supplementary Material

Supp.Table

Supp.Information
