# Supplementary material for: Antibiotic perturbation of the murine gut microbiome introduces inter-individual susceptibility to arsenic
Source: Toxicology. Author manuscript; Available in PMC 2021 Jun 15. (PMC8204511; doi:10.1016/j.tox.2021.152798)
Supplement: Supp.Table [file NIHMS1696844-supplement-Supp_Table.docx]

**Supplemental Information: Health Assessment for Arsenic Exposure**

**At the outset of arsenic exposure:**

***Once daily observation will occur until clinical signs begin to develop such as:***

***Stage 1 disease symptoms***

• rough hair coat

• hunched posture

• 10% weight loss

• observable decrease in body condition

• dehydration (decreased skin turgor, weight loss, dull or sunken eyes)

**Once any of these signs develop, observations will increase to twice daily (approx. 6 hours apart):**

***Stage 2 disease symptoms***

*Additional signs of increased morbidity will be recorded, including:*

• decreased activity/exploration

• decreased nest building

• decreased intake of food/water

• squinting, hunched posture

• piloerection/rough hair coat

**Signs that will require immediate euthanasia include:**

***Stage 3 disease symptoms***

• impaired ambulation/reluctance to move (moves only with manual stimulation)

• difficult labored breathing

• inability to remain upright/ataxia

• tremors/shivering

• emaciation

• weight loss of 25%* (if this is the only clinical sign, a veterinary consult may allow the experiment to continue)

• sizeable abdominal distension/ascites

• severe hunched posture, significant squinting/closure of eyes/nose bulge

• persistent recumbency
