# Supplementary material for: Antibiotic perturbation of the murine gut microbiome introduces inter-individual susceptibility to arsenic
Source: Toxicology. Author manuscript; Available in PMC 2021 Jun 15. (PMC8204511; doi:10.1016/j.tox.2021.152798)
Supplement: Supp.Information [file NIHMS1696844-supplement-Supp_Information.docx]

**Supplemental Table 1. Mortality Experiment Details**

| **Exp #** | **Sex** | **# Mice/Cage** | **Age (weeks)** | **Treatment** |
| --- | --- | --- | --- | --- |
| 1 | F | 5 | 10 | Antibiotic-Arsenic |
|  | F | 4 | 10 | Antibiotic-Arsenic |
|  | M | 5 | 10 | Antibiotic-Arsenic |
|  | M | 5 | 10 | Antibiotic-Arsenic |
| 2 | F | 4 | 7 | Arsenic-only |
|  | M | 5 | 7 | Arsenic-only |
| 3 | F | 5 | 7 | Antibiotic-only |
|  | M | 5 | 7 | Antibiotic-only |
| 4 | M | 5 | 13 | Antibiotic-Arsenic |
|  | M | 5 | 13 | Sham-Arsenic |
| 5 | F | 5 | 10 | Sham-Arsenic |
|  | F | 5 | 10 | Antibiotic-Arsenic |
|  | F | 5 | 10 | Antibiotic-Arsenic |
|  | M | 5 | 10 | Antibiotic-Arsenic |
|  | M | 5 | 10 | Antibiotic-Arsenic |
| 6 | F | 4 | 9 | Antibiotic-Arsenic |
|  | F | 4 | 9 | Sham-Arsenic |
|  | M | 5 | 9 | Antibiotic-Arsenic |
|  | M | 5 | 9 | Sham-Arsenic |
| 7 | F | 5 | 9 | Antibiotic-Arsenic |
|  | F | 5 | 9 | Arsenic-only |
|  | M | 5 | 9 | Antibiotic-Arsenic |
|  | M | 5 | 9 | Arsenic-only |
| 8 | M | 3 | 9 | Sham-Arsenic |
|  | M | 5 | 9 | Antibiotic-Arsenic |
|  | F | 5 | 9 | Antibiotic-Arsenic |
|  | F | 5 | 9 | Antibiotic-Arsenic |
| 9 | F | 5 | 9 | Antibiotic-Arsenic |
|  | F | 5 | 9 | Antibiotic-Arsenic |
| 10 | M | 5 | 9 | Antibiotic-only |
